# How does arch form and interproximal contact size affect the 3D displacements and rotations of teeth: a finite element analysis

**DOI:** 10.1590/2177-6709.28.6.e232381.oar

**Published:** 2024-01-05

**Authors:** Andrea ÁLVAREZ, Santiago Alberto CORREA, Peter H. BUSCHANG, Samuel I. ROLDÁN

**Affiliations:** 1 Universidad CES (Medellin, Colombia).; 2 Universidade EAFIT, Escuela de Ingenieria (Medellin, Colombia).; 3 Texas A&M University Baylor College of Dentistry, Orthodontics (Dallas/Texas, United States).

**Keywords:** Crowding, Arch form, Interproximal contacts, Anterior component of force, Interproximal enamel reduction

## Abstract

**Objective:**

The objective of this study was to determine how arch form and interproximal contact size displace mandibular teeth subjected to an anterior component of force (ACF).

**Methods:**

Nine finite element models (FEM) of the mandibular arch were developed using Ansys® v. 16.0 software. They were designed to evaluate the effects of three arch forms (triangular, oval, and square) and three contact sizes (point-to-point, 1 mm diameter, and 2 mm diameter). All nine models were subjected to an ACF of 53.8 N (5486 gm). Three-dimensional tooth rotations and displacements of the mandibular teeth were evaluated, from the right first molar to the left first molar.

**Results:**

Arch form had a greater effect on tooth movements than contact size. Triangular arches and point-to-point contacts produced the greatest displacements and rotations of teeth. Oval arches with 2 mm wide interproximal contact points showed the greatest stability. The right first premolar showed the greatest displacements in all of the models.

**Conclusions:**

Arch form and contact size affect interproximal tooth stability. Teeth are least stable in narrow arches with point-to-point interproximal contacts, and most stable in wider arches with larger contacts.

## RESUMO

**Objetivo:**

O objetivo deste estudo foi determinar como o formato da arcada e o tamanho do contato interproximal deslocam os dentes inferiores submetidos a um componente anterior de força (CAF).

**Métodos:**

Nove modelos de elementos finitos (MEF) da arcada inferior foram desenvolvidos utilizando o *software* Ansys® v. 16.0. Eles foram projetados para avaliar os efeitos de três formatos de arcada (triangular, oval e quadrado) e três tamanhos de contato interproximal (ponto a ponto, 1 mm de diâmetro e 2 mm de diâmetro). Todos os nove modelos foram submetidos a um CAF de 53,8 N (5486 gm). Foram avaliados tridimensionalmente as rotações dentárias e os deslocamentos dos dentes inferiores, do primeiro molar direito ao primeiro molar esquerdo.

**Resultados:**

A forma da arcada teve um efeito maior no movimento dos dentes do que o tamanho do contato interproximal. Arcadas triangulares e contatos ponto a ponto produziram os maiores deslocamentos e rotações dos dentes. Arcadas ovais com pontos de contato interproximal de 2 mm de largura apresentaram maior estabilidade. O primeiro pré-molar direito apresentou os maiores deslocamentos em todos os modelos.

**Conclusões:**

O formato da arcada e o tamanho do contato interproximal afetam a estabilidade dos dentes. Os dentes foram menos estáveis nas arcadas estreitas com contatos interproximais ponto a ponto, e mais estáveis nas arcadas mais largas com contatos maiores.

## INTRODUCTION

Dental malalignment is widely studied due to its impact on aesthetics and quality of life.^1^ According to the third National Nutrition Survey (NHANES III), 30% of the US population has significant crowding and 15% has severe crowding. High prevalence of malalignment has also been reported in other populations. Due to the number of individuals affected, malalignment is considered an endemic condition in modern society.^2^

While various factors have been associated with malalignment, including tooth size^3^ ^,^ ^4^ and dental arch size,^4^ ^-^ ^6^ the basic problem after the permanent teeth have erupted is contact displacements (i.e., contact “slippage”).^7^ In addition to vertical growth and associated dental eruption,^7^ ^-^ ^9^ the anterior component of force (ACF) causes teeth to move and contacts to slip.^4^ ^,^ ^7^ ^,^ ^8^ As described by Southard et al.^10^, the ACF is the horizontal component of bite force associated with the axial inclinations of the teeth. The existence of the ACF, as well as its association with late crowding are well established.^10^ ^-^ ^13^ Anything that displaces teeth anteriorly can cause instability at interproximal contacts and malalignment. This can explain why patients who received post-treatment interproximal restorations showed significantly greater increase in incisor irregularity after orthodontic treatment than those without restorations.^6^

While interproximal contact size and shape is expected to be related to mandibular malalignment, this association have not been well studied. Ihlow et al^14^, who used Plexiglass cylinder plates with teeth to evaluate contacts, concluded that concave-convex contacts are more stable than point-to-point contacts. It has been suggested that interproximal enamel reduction, which increases contact size, decreases long-term malalignment by up to 25%.^15^

Theoretically, teeth in narrow arches should be less stable than teeth in wide arches. Myser et al^6^ reported that post-treatment interdental angles, which provided indirect measures of arch shape, were related to post-treatment incisor irregularity and anterior tooth-size-arch-length discrepancies (TSALD) changes. They also showed that the mandibular canines and lateral incisors exhibited the smallest inter-contact angles and the greatest post-treatment contact discrepancies, further supporting the relationship between arch shape and malalignment. This supports previous associations between incisor irregularity increases between 13 and 31 years of age and arch shape.^16^ Importantly, clinical studies such as these are not able to control for the various factors that could explain the changes that have occurred.

Due to the possible confounding effects of individual differences in tooth morphology, bite forces, material properties of bone, teeth and soft tissues, and craniofacial morphology, clinical studies are not well suited to test the isolated effects of arch form and contact size. Mathematical modeling makes it possible to overcome such difficulties. Due to their reliability and ability to analyze biological systems, finite element method (FEM) has been previously used to evaluate the craniofacial complex.^17^ Moreover, FEM makes it possible to assess complex clinical problems without putting patients at risk. Most importantly, FEM makes it possible to simplify the morphology of structures in order to evaluate their effects independently of other possible confounders.

Thus, the purpose of the present study was to subject a simplified FEM model of the mandibular dentition to a biologically realistic ACF. A simplified model was used to isolate the effects of contact size and arch form on tooth movements. Due to the potential interaction, it is essential to control for arch shape when evaluating contact size, and vice versa. A simplified model also provides greater numerical stability.

## MATERIAL AND METHODS

Nine mandibular FEM models were developed to evaluate three lower arch forms (triangular, oval, and square) and three interproximal contact sizes (point-to-point, 1 mm, and 2 mm in diameter). The geometry of the models was created using PTC^®^ Creo v. 3.0 software (Needham, USA).

### TOOTH SIZE, CROWN SIZE, AND ALVEOLAR BONE

**Figure 1:**
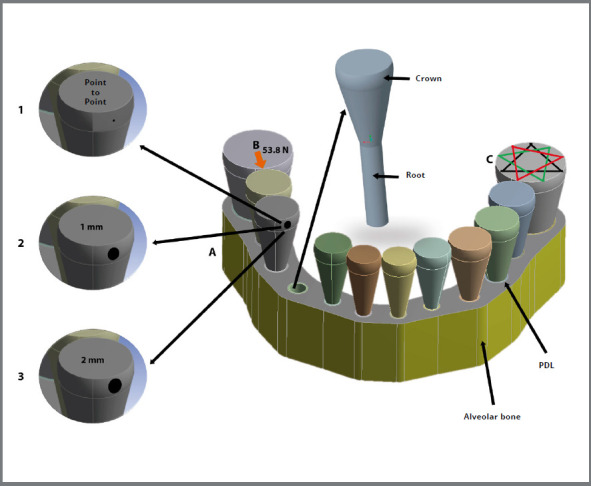
A = Contour condition given by alveolar bone. B = Point of application of force to the right first molar. C = Multiple triangles, in order to calculate rotations and displacements. 1 = Point to point contact, 2 = 1 mm contact, 3 = 2 mm contact.

To simplify the models, the crowns and roots of the teeth were modeled as cones (Fig 1). Using standardized cones made it possible to eliminate the confounding effects of different tooth morphologies. The models were generated to approximate actual crown widths, teeth lengths and tooth heights.^18^ The periodontal ligament was modeled for each of the roots to be 0.25 mm thick.^17^ To further simplify the models, all teeth were oriented at 90º to the basal bone, to minimize the bias that different angulations could introduce^19^.

The cortical bone was modeled to gradually increase in thickness from anterior to posterior, thereby more closely representing actual anatomical bone morphology.^20^ The lamina dura around the roots of the teeth was modeled to be 1.7 mm thick.^21^

### MANDIBULAR ARCH FORMS AND DIMENSIONS

The three arch forms and dimensions of the mandibular arch (triangular oval, square) were based on the beta function proposed by Braun et al.^22^:

$$Y=3.0314*D*{\left[\frac{X}{W}+\frac{1}{2}\right]}^{0.8}{\left[\frac{1}{2}-\frac{X}{W}\right]}^{0.8}$$ (1)

**Figure 2:**
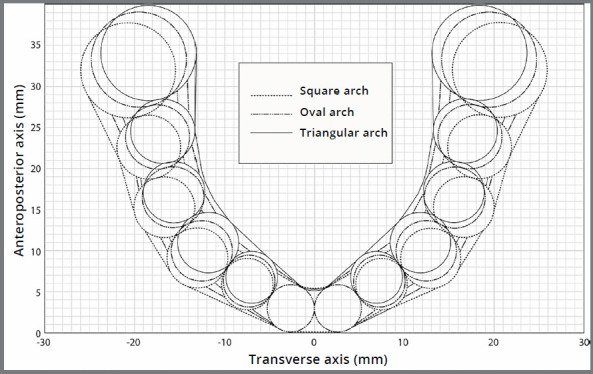
Square, Oval and Triangular arch forms, based on beta function replaced with values reported by Nojima et al.^23^

In the formula above, W represents the distance between the disto-buccal cusps of the second molars, D represents the perpendicular distance between the anterior point between the central incisors and the line joining the disto-buccal cusps of the second molars, and X represents any point in the transverse axis (Fig 2). The three arch forms were estimated using the inter-canine widths, inter-molar widths and depths reported for Caucasians (Table 1).^23^ The most vestibular point of every tooth was aligned along the inner portion of each arch form.

**Table 1:** Intra-arch distances and depths (in mm) in the three arch forms.

	Inter lateral incisor width	Inter canine width *	Inter first premolar width	Inter second premolar width	Inter molar width*	Inter lateral incisor depth	Inter canine depth*	Inter first premolar depth	Inter second premolar depth	Inter molar depth*
Triangular	20.2	28.4	37.0	43.3	47.9	2.6	6.8	11.8	17.7	27.5
Oval	20.3	29.4	38.0	44.7	49.8	2.3	6.1	14.6	20.5	27.0
Square	20.6	29.6	39.3	46.5	52.2	2.1	5.3	10.5	16.1	26.2

* Source: Nojima et al.^23^ (2001).

### INTERPROXIMAL CONTACTS

The interproximal contacts were simulated by a non-linear surface-to-surface contact algorithm. Friction was not taken into account, due to its highly non-linear behavior, which caused the algorithm to diverge. An algorithm determined the point-to-point contact based on where the convex surfaces of both teeth touched. For 1mm and 2mm diameter contacts, two flat surfaces were created manually (Fig. 1). Importantly, the contacts were purposefully the same for all the teeth, so that their effects could be assessed independently of arch shape. The three arch forms (triangular, oval, and square) had the same perimeter, but arch perimeter differed depending on contact size. Arch perimeter was slightly less for 2 mm than 1 mm contacts, and less for point-to-point than 1 mm contacts.

### FINITE ELEMENT MESH

The models were exported to the Ansys^®^ v. 16.0 software (Pittsburgh, USA) and a mesh was constructed. The mesh consisted of tetrahedral elements that had 10 elastic linear nodes that were 0.7 mm long for alveolar bone and teeth, and 0.1 mm long for the periodontal ligament. The mesh was validated using the “h” method, in which the sizes of the elements were progressively reduced until variation of the displacements reached 5%.

### MECHANICAL PROPERTIES

Specific mechanical properties were assigned to the teeth, bone, and periodontal ligament (PDL) (Table 2). The cortical alveolar bone was divided into three portions, including the symphyseal, canine-premolar, and molar regions. It had orthotropic properties in the bucco-lingual, gingival-incisal, and mesio-distal directions.^24^ The periodontal ligament was considered linearly elastic and isotropic, with a constant thickness of 0.25 mm.^17^ Teeth were modeled to be composed of isotropic dentin.^25^

**Table 2:** Mechanical Properties utilized to create the models.

Region/Structure	Modulus of elasticity (MPa)			Shear stiffness modulus (MPa)			Poisson Coefficient		
	E1	E2	E3	G12	G13	G23	V12	V13	V23
Mandibular Cortical Bone*									
									
Molar bone*	19450	13600	10250	6250	5900	4150	0.34	0.29	0.21
Canine-premolar bone*	25500	14400	10200	6250	5050	3450	0.15	0.21	0.31
Symphysis bone*	22400	14200	10650	6000	4850	3650	0.215	0.28	0.30
Periodontal Ligament**	0.68	0.68	0.68	0.23	0.23	0.23	0.49	0.49	0.49
Dentin***	16300	16300	16300	6200	6200	6200	0.25	0.25	0.25

* Source: Schwartz-Dabney and Dechow^24^. ** Source: Provatidis et al^17^. *** Source: Kinney et al^25^

### LOADS AND CONTOUR CONDITIONS

To simulate the ACF, a force of 53.8 N (5486 gm) was applied between the first right molar and the first right premolar, as reported by Southard et al.^10^ The force was applied in a mesial direction, perpendicularly to the interproximal surfaces, at the contact points or contact planes (Fig 1). Displacements were fixed perpendicular to the periphery of the cortical bone (buccal and lingual) with 6 degrees of freedom.

### DATA COLLECTION

The displacements and rotations of each tooth were estimated in the transverse (x), anteroposterior (y) and vertical (z) planes. These estimates were determined by equilateral triangles drawn on the occlusal surface of each tooth. The number of triangles per tooth varied from 14 to 28, depending on the tooth size. The displacements in the x, y and z planes were obtained by averaging the displacements of triangles’ nodes; the rotations were obtained by averaging the product of the two vectors that defined each triangle (Fig 1). The magnitude and direction of the resulting vectors were displayed by the number, thicknesses, curvature and colors of arrows, with red representing the highest and blue, the smallest magnitudes (Fig 4). 

## RESULTS

**Figure 3:**
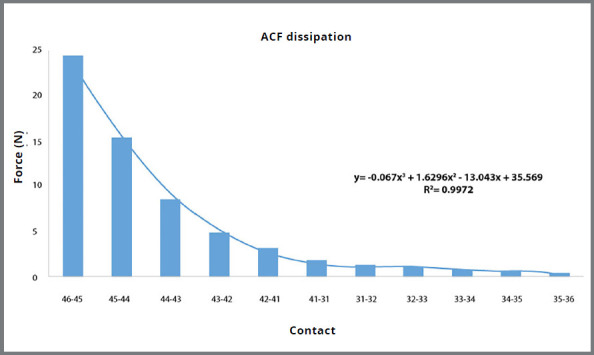
Average reduction of Anterior Component of Force (ACF) from the 46-45 contact to the contralateral side of the arch in the 9 models simulated. “y” is the function of the curve reduction. R^2^ is the correlation between the 9 models for each interproximal contact.

All nine models showed similar force dissipation patterns, regardless of arch shape or contact size (Fig. 3). The ACF decreased regularly from the right molar/premolar contact to the contralateral left molar/premolar contact. There was a 65% decrease in the ACF between the molar/premolar contacts and canine/lateral contacts on the side in which the force was applied, and a 93% decrease between the molar/premolar contact and central incisors. 

No significant differences in displacement or rotation were observed between the nine models in the vertical (z) plane. In contrast, the transverse (x) and sagittal (y) planes showed consistent patterns of displacement and rotations depending on contact size and arch shape. 

**Figure 4:**
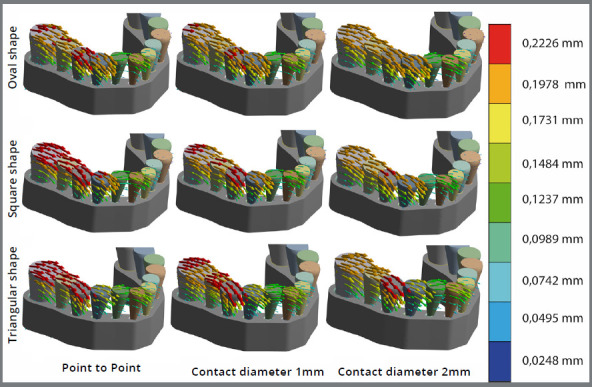
Displacements and rotations of the models. Red represents the greatest displacement, and dark blue represents the smallest displacement. Rotations are represented by the combination of colors in the same tooth (e.g. right molar of the oval shaped arch with point-to-point contacts rotates more than the right molar of the oval shaped arch with 1 mm contacts, but less than the first premolar in the arch with 1 mm contacts.

Arch shape had a greater effect on tooth displacements and rotations than contact size (Fig. 4). The first molars and premolars showed the least displacement in oval arches and the greatest displacements in triangular arches. The first premolars showed less rotation in oval and square arches than in triangular arches (see curvature of the red arrows in Fig 4). The canines rotated distolingually-mesiobucally in all three arch forms, due to the force vectors of the first premolars, and this pushed the laterals mesially. The lateral and central incisors showed similar patterns of rotation and displacement, although the amounts were higher in triangular arches. 

Models with 2 mm wide contact areas were more stable than 1 mm contacts, which in turn were more stable than point-to-point contacts (Fig 4). Point-to-point contacts showed the greatest displacements, regardless of arch form. Displacements of the first molars and the second premolars were greater in arches with point-to-point contacts than in arches with 1 mm contacts, which were in turn greater than displacements in arches with 2 mm contacts. Although differences were higher for every type of contact in triangular arches, when compared with oval and squared arches, the first premolar showed the same pattern of displacement as the molars and second premolars. Canines and incisors showed similar patterns of displacement and rotation, regardless of the type of interproximal contact.

## DISCUSSION

The ACF produced tooth displacements in all three planes of space, even on the contralateral side (Fig. 3). This was caused by the forces transmitted through the interproximal contacts. Multiple studies that have evaluated the ACF show that it continues through to the opposite side of the arch.^10^ ^,^ ^11^ ^,^ ^13^ The interseptal fibers probably play a role in retaining the teeth, while the force propagates across the midline.

The ACF decreased progressively from the point of force application to the incisors on the contralateral side. Similar reductions of the ACF have been previously reported.^10^ ^,^ ^11^ ^,^ ^13^ The force between the lateral and canine on the contralateral side was 142.7 gF, representing a decrease of 96%, which is sufficient to cause contacts to slip. Forces as small as 15.9 gF can cause dental movements.^26^ The forces propagating through the arch would have been greater if the teeth had been mesially inclined, as they are naturally^19^; and greater strains would have been produced due to the increased distances to the center of resistance of each tooth, which produces a larger moment.

The square and oval arches showed less displacement of the molars and premolars than triangular arches. The arches presented width differences (triangular arches being the narrowest) of the posterior teeth, which were greater in the back than in the front (Fig. 2). As such, the same ACF might be expected to have less resistance from the teeth in triangular arch forms, allowing greater displacements of the molar and premolars (Fig. 4), as shown in the present study. Triangular arches showed the highest slippage of contacts between the canine and first premolars, regardless of the type of contact. Higher interproximal strains are expected in narrower arches, particularly at the canine-lateral and first premolar-canine contacts, where occurs the biggest curvature of the dental arch^14^. Assuming the same bicondylar width, narrow arches have a larger moment arm between the working condyle and the bite point when biting unilaterally. A narrow arch will have a bite point closer to the midline, which increases the bite force moment arm. This produces higher forces on the interproximal contacts, which could increase the ACF. It should be emphasized that, in terms of stability, the present study showed that arch form was more important than contact size. Differences in arch shape could explain why post-treatment stability is greater in non-extraction than extraction cases.^6^

The contacts between canines and first premolars showed the greatest slippage in all three arch forms, with the largest displacements occurring in triangular arches with point-to-point contact. It has been previously shown that the contacts between the mandibular canines and laterals are the most displaced (i.e. largest contact displacements), regardless of the amount of crowding present.^2^ ^,^ ^9^ However, these studies only evaluated contacts between the six anterior teeth. The present study found the greatest displacement between canines and first premolars, probably because of the marked curvature in the area were the canines are located. 

Larger areas of interproximal contact produced less displacement and rotation than point-to-point contacts, regardless of the arch form. The FEM models with 2 mm contacts were the most stable, followed by 1 mm contacts and point-to-point contacts, respectively (Fig 4). Prehistoric arches rarely show evidence of malalignment, most likely due to the natural wear patterns produced by their fibrous diet.^27^ Bitewing radiographs of present day young adults also show natural wear patterns, albeit to a lesser degree, with thinner enamel on the mesial surfaces, which tend to be concave.^28^ The present findings corroborate the study done with Plexiglass models showing that arches with point-to-point contact are less stable than arches that had greater surface area.^14^ The clinical significance of this relation pertains to the increase in malalignment commonly observed in untreated individuals.^2^ Broader contacts between the anterior teeth might be expected to enhance their long-term stability, suggesting that interproximal enamel reduction (IPR) can enhance stability. It has been previously suggested that IPR reduces post-treatment relapse.^15^

As a FEM, this study is limited in its ability to model biological phenomenon, due primarily to the geometric simplifications and material properties used. The actual crown geometry of teeth might be expected to produce different displacement and rotational patterns, especially for the posterior teeth, making it difficult to isolate the effects of arch shape and contact size. The present models show these effects when other potential sources of variation have been controlled. Properties of the periodontal ligament were considered isotropic, when in fact they are anisotropic. A wide range of anisotropic values have been reported.^17^ Although the PDL anisotropical modeling more closely resembles its actual behavior, it would produce greater displacement and rotation, because the center of resistance of the teeth is located more apical than if it was modeled isotropically. The expected difference between both type of modeling should be less than 6%.^17^

The instantaneous rotations and displacements demonstrated in the present study were small, because they were caused by a single load. In daily life, individuals typically experience at least three episodes of chewing per day, each with 15 minutes in duration, at a chewing rate of more than 60 cycles per minute.^29^ This is equivalent to 2,700 chewing cycles per day. This can result in tooth movements that accumulate throughout the day or part of the day, as previously shown for daily tooth eruption.^30^ Not much movement would be required to displace and slip contacts, especially if they are point-to-point in narrow arches. Clinically, the present results indicate that any form of treatment that maintains tight interproximal contacts (e.g. power chain with brackets or aligners) from first molar to first molar should be reconsidered in patients with triangular arches.

## CONCLUSIONS

1. The anterior component of force causes slipping between interproximal contacts, resulting in the displacement and rotation of teeth, with the first premolars showing the greatest movements.
2. In terms of both rotation and displacement of teeth, triangular arch forms were the least stable, while oval and square arch forms were the most stable.
3. Teeth with larger interproximal contacts showed less rotation and displacement than teeth with point-to-point contacts.
4. Arch shape had a greater effect on the rotation and displacements of teeth than contact size.
